# Complete chloroplast genome sequence of *Patrinia saniculifolia* hemsl. (Disacales: Caprifoliaceae), an endemic plant in Korea

**DOI:** 10.1080/23802359.2017.1419098

**Published:** 2017-12-27

**Authors:** Eun-Hee Jung, Chae Eun Lim, Byoung Yoon Lee, Suk-Pyo Hong

**Affiliations:** a National Institute of Biological Resources(NIBR), Incheon, Republic of Korea;; b Department of Biology, Kyung Hee University, Seoul, Republic of Korea

**Keywords:** *Patrinia saniculifolia*, endemic plant, complete chloroplast genome, Caprifoliaceae, Dipsacales, phylogenetic analysis

## Abstract

*Patrinia saniculifolia* Hemsl. is a Korean endemic plant belongs to the family Caprifoliaceae s. l. In this study, we report the complete chloroplast genome of *P. saniculifolia*. The chloroplast genome was 153,775 bp with LSC (87,529 bp) and SSC (17,236 bp) regions, separated by two IRs regions 23,806 bp, and overall GC content was 38.48%. It contains total of 111 genes, including 78 protein-coding genes, 29 tRNA genes, and 4 rRNA genes.

*Patrinia saniculifolia* Hemsl. is a Korean endemic plant and a perennial herb. *Patrinia saniculifolia* is an alpine plant of Korea with the exceptions of Jeju lsland and Ulleung Island. Morphological characteristics of *P. saniculifolia* apart from other species in the genus are palmated basal leaves, long spur on flowers, and oblong bracteoloe of fruits etc. The genus *Patrinia* Juss. belongs to the family Caprifoliaceae s. l. (subfamily Valerianoideae) in the order Dipsacales. The subfamily Valerianoideae has been recognized as the family previously. The traditional family Valerianaceae comprised ca. 400 species, distributed worldwide without Australia and divided into three tribes: Triplostegieae Höck, Patrinieae Höck, and Valerineae Höck (Graebner 1906). Molecular data strongly support that the tribe Patrinieae, comprising *Patrinia* and *Nardostachys* DC. is a basal group within the family (Bell et al. 2001; Donoghue et al. 2001; Pyck et al. 2002; Bell 2004; Hidalgo et al. 2004). The genera *Patrinia* and *Nardostachys* are mainly restricted in Asia and considered as important medicinal plants (Sahu et al. 2016; Xiru et al. 2017).

*Patrinia saniculifolia* was collected from Kangwon province in Korea. Voucher specimen (NIBRVP0000642096) was deposited in the herbarium (KB) of the National Institute of Biological Resources. Total genomic DNA was extracted from young leaves and sequenced using the Illumina MiSeq platform (Illumina Inc., San Diego, CA). Obtained high quality paired-end reads of about 2.4 Gb were newly assembled to characterize chloroplast genome, as described previously (Kim et al. 2015).

The complete chloroplast genome is a circular DNA molecule of 153,775 bp in length (GenBank Accession MG517444) and has typical quadripartite structure consisting of large single copy (LSC) region of 88,927 bp, small single copy (SSC) region of 17,236 bp, and a pair of inverted repeats (IRa and IRb) of 23,806 bp. A total of 111 genes were annotated in the chloroplast genome, which included 78 protein-coding genes, 29 transfer RNA genes, and 4 ribosomal RNA genes. Among the genes, 15 were duplicated in IR regions. Overall GC contents of chloroplast genome are 38.48%.

Phylogenetic relationships between *P. saniculifolia* and 15 taxa (five species from Adoxaceae, three species from Caprifoliaceae, each two species from Apiales, Asterales, and Aquifolilales, and two outgroup species) were inferred based on a Maximum-Likelihood (ML) tree of 57 common chloroplast protein-coding gene sequences (Figure 1). The ML tree by MEGA 6.0 (Tamura et al. 2013) showed that *P. saniculifolia* formed a distinct group with other two species in the family Caprifoliaceae and had a sister relationship with species in the family Adoxaceae (Figure 1). Complete cp genome of *P. saniculifolia* provides valuable data for resolving evolutionary and phylogenetic issues in the genus *Patrinia* and the family Carprifoliaceae, and can be used as cp DNA markers for species classification.

**Figure 1.**
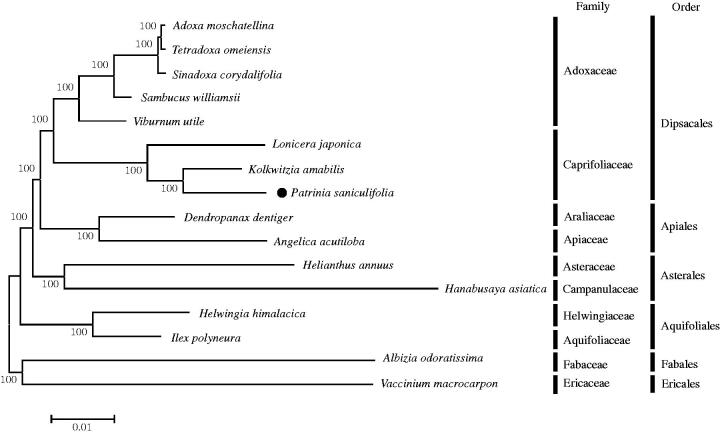
Maximum Likelihood tree based on the chloroplast protein coding genes of 16 taxa including *Patrinia saniculifolia* and two outgroup taxa. Common chloroplast protein coding gene sequences were aligned using MAFFT (http://mafft.cbrc.jp/alignment/server/index.html) and subjected to generating Maximum Likelihood phylogenetic tree by MEGA 6.0 (Tamura et al. 2013). The bootstrap support values (>50%) from 1,000 replicates are indicated in the nodes. Chloroplast genome sequences used for this tree are: *Adoxa moschatellina*, NC_034792; *Albizia odoratissima* (outgroup), NC_034987; *Angelica acutiloba*, NC_029391; *Dendropanax dentiger*, NC_026546; *Hanabusaya asiatica*, NC_024732; *Helianthus annuus*, NC_007977; *Helwingia himalacica*, KX434807; *Ilex polyneura*, KX426468; *Kolkwitzia amabilis*, NC_029874; *Lonicera japonica*, NC_026839; *Sambucus williamsii*, NC_033878; *Sinadoxa corydalifolia*, NC_032040; *Tetradoxa omeiensis*, NC_034793; *Vaccinium macrocarpon*, JQ757046 (outgroup); *Viburnum utile*, NC_032296; *Patrinia saniculifolia*, MG517444.

## References

[CIT0001] Bell CD, Edwards EJ, Kim ST, Donoghue MJ. 2001 Dipsacales phylogeny based on chloroplast DNA sequences. Harv Pap Bot. 6:481–499.

[CIT0002] Bell CD. 2004 Preliminary phylogeny of Valerianaceae (Dipsacales) inferred from nuclear and chloroplast DNA sequence data. Mol Phylogenet Evol. 31:340–350. 15019629 10.1016/j.ympev.2003.07.006

[CIT0003] Donoghue MJ, Eriksson T, Reeves PA, Olmstead RG. 2001 Phylogeny and phylogenetic taxonomy of Dipsacales, with special reference to *Sinadoxa* and *Tetradoxa* (Adoxaceae). Harv Pap Bot. 6:459–479.

[CIT0004] Graebner P. 1906 Die Gattungen der natürlichen Familie der Valerianaceae. Bot Jahrb Syst. 37:464–480.

[CIT0005] Xiru H, Luan F, Zhao Z, Ning N, Li M, Jin L, Chang Y, Zhang Q, Wu N, Huang L. 2017 The Genus *Patrinia*: a review of traditional uses, phytochemical and pharmacological studies. Am J Chin Med. 45:637–666. 28595500 10.1142/S0192415X17500379

[CIT0006] Hidalgo O, Garnatje T, Susanna A, Mathez J. 2004 Phylogeny of Valerianaceae based on *mat*K and ITS markers, with reference to *mat*K individual polymorphism. Ann Bot. 93:283–293. 14988097 10.1093/aob/mch042 PMC4242203

[CIT0007] Kim K, Lee SC, Lee J, Yu Y, Yang K, Choi BS, Koh HJ, Waminal NE, Choi HI, Kim NH, et al 2015 Complete chloroplast and ribosomal sequences for 30 accessions elucidate evolution of *Oryza* AA genome species. Sci Rep. 5:15655 26506948 10.1038/srep15655 PMC4623524

[CIT0008] Pyck N, Lysebetten VA, Stessens J, Smets E. 2002 The phylogeny of Patrinieae sensu Graebner (Valerianaceae) revisited: additional evidence from ndhF sequence data. Plant Syst Evol. 233:29–46.

[CIT0009] Sahu R, Dhongade HJ, Pandey A, Sahu P, Sahu V, Patel D, Kashyap P. 2016 Medicinal Properties of *Nardostachys jatamansi* (A Review). Orient J Chem. 32:859–866.

[CIT0010] Tamura K, Stecher G, Peterson D, Filipski A, Kumar S. 2013. MEGA6: Molecular Evolutionary Genetics Analysis Version 6.0. 10.1093/molbev/mst197 PMC3840312 24132122

